# The Role of the Food and Drug Administration in Drug Development: On the Subject of Proarrhythmia Risk

**DOI:** 10.19102/icrm.2020.110103

**Published:** 2020-01-15

**Authors:** Munveer Thind, Peter R. Kowey

**Affiliations:** ^1^Division of Cardiology, Lankenau Heart Institute, Wynnewood, PA, USA; ^2^Thomas Jefferson University, Philadelphia, PA, USA

**Keywords:** Approval, development, drug, FDA, proarrhythmia, safety

## Abstract

The Food and Drug Administration (FDA) is responsible for the regulation of the pharmaceutical industry in the interest of protecting public health. The aim of this review was to outline the evolution and current role of the FDA in the development and approval of new drugs. Additionally, we describe current assessments of proarrhythmia risk to illustrate recent FDA initiatives intended to harness information technology to modernize the regulatory process. In order to identify the literature required to produce this review, search tools such as PubMed and Google Scholar were used to locate relevant web pages and articles. The job of the FDA is not only to ensure that high standards for drug efficacy and safety are applied to products available to American consumers and patients but also to balance the lengthy, costly process of maintaining these standards against the pressure to provide access to effective treatments earlier and without surplus expenditures. In order to provide expedited access to the newest effective therapies for critically ill patients in the safest way possible, the FDA has developed several accelerated pathways to fast-track drug approval. Through partnerships with industry and academic institutions, research is being conducted into how information technology can be integrated into the drug development process to improve its cost-effectiveness.

## Introduction

The proportion of Americans taking at least one prescription drug has been steadily increasing and approaches 50% of the population,^1^ and the projected number of prescriptions expected to be filled by Americans in 2019 was estimated to be 4.25 billion.^2^ The United States (US) Food and Drug Administration (FDA) is the organization responsible for protecting public health by ensuring that the drugs available on the US market meet certain standards for both safety and efficacy.^3^ This review was put together to inform both the public and prescribers of the key aspects of the process during which a chemical entity in a laboratory is developed into a therapeutic drug approved by the FDA and made available to consumers.

## Organization

**Figure 1:**
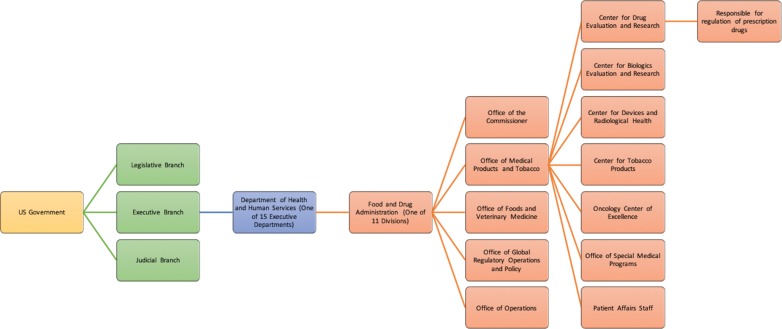
Illustration of the position of the FDA in the US governmental hierarchy.

The FDA is a division within the US Department of Health and Human Services and is made up of five directorates, including the Office of Medical Products and Tobacco. The Center for Drug Evaluation and Research (CDER), a subsection of the Office of Medical Products and Tobacco, is the primary body responsible for the regulation of over-the-counter and prescription drugs^4^ **(Figure 1)**.

## History and legislative landmarks

**Figure 2:**
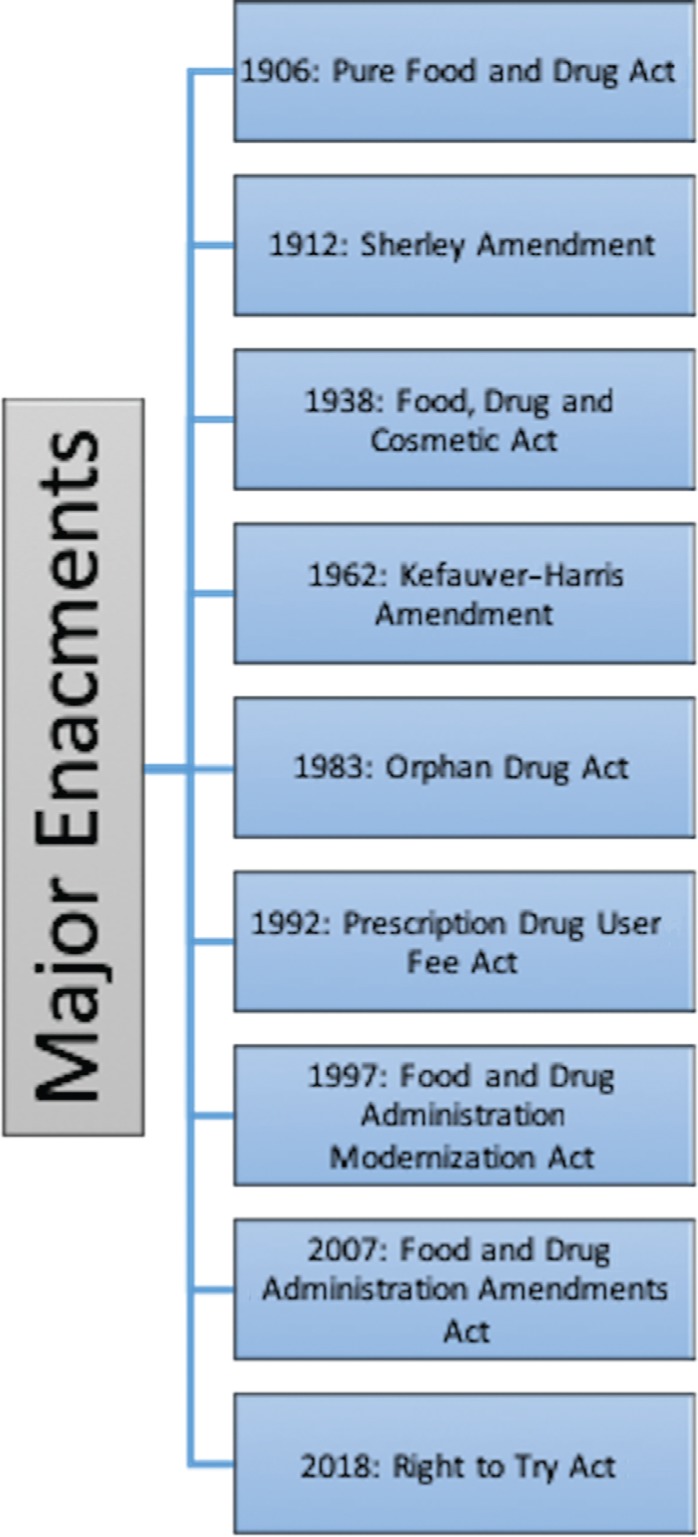
Timeline of major landmarks in the evolution of drug regulation efforts in the US.

Until the early 1900s, drug products were able to be sold with false labels describing unregulated ingredients as well as unsubstantiated claims about their therapeutic merit. In 1906, Congress passed the first major consumer protection law called the Pure Food and Drug Act,^5^ which prohibited the interstate sale of misbranded food and drugs with regard to their ingredients and purity **(Figure 2)**. This was followed in 1912 by the Sherley Amendment, which outlawed the labeling of drugs with false therapeutic claims.^6^ These laws were enforced by the Bureau of Chemistry in the Department of Agriculture, which became the FDA in 1930.^7^

The next major landmark in drug regulation came in 1938, when the Food, Drug, and Cosmetic (FD&C) Act was passed.^8^ This was prompted by the events of 1937, during which 105 people died as a result of taking elixir sulfanilamide. A Tennessee drug company, S.E. Massengill Co., developed the drug by formulating sulfanilamide, the first sulfa antimicrobial, with diethylene glycol in order to produce a liquid form of the antibiotic.^9^ However, diethylene glycol, a derivative of ethylene glycol, which is now used in antifreeze formulations, is toxic to humans and causes metabolic acidosis, nephrotoxicity, and neurotoxicity. The subsequent public outcry led to the passage of the FD&C Act, which included many new provisions focused on ensuring that a drug’s safety was established prior to a product being released on the market.

The next major enactment came in 1962, when the Kefauver–Harris Amendment was passed.^7^ This bill required drug manufacturers to prove that their product was both effective and safe, prior to release on the market, and to report any adverse effects observed during the postmarketing period to the FDA. It also stipulated that the drug’s effectiveness be demonstrated in well-controlled clinical trials and that the patients in those trials must provide informed consent prior to their inclusion. Additionally, it allowed the FDA to control prescription drug advertising in order to ensure the accurate reporting of side effects.^10^ This amendment enabled the FDA to require “adequate and well-controlled investigations” demonstrating substantial evidence of efficacy^11^ and established the blueprint for conducting randomized controlled trials, which remain the benchmark for demonstrating drug efficacy and safety today.

In 1983, the Orphan Drug Act was passed and provided incentives like market exclusivity and tax credits to promote the development of drugs aimed at treating rare diseases.^12^ Similar legislation has since been adopted in Japan and the European Union.^13^

In 1992, the Prescription Drug User Fee Act was passed by Congress. This allows the CDER to collect fees from drug companies to be used to boost the resources committed toward the approval process of a specific drug. In return, agreed stages of the approval process must be completed by deadlines determined at the time at which the fee is collected. This procedure helps the FDA to bring drugs to the market in a timely fashion without sacrificing the careful review process.^14^

In 1998, the Adverse Event Reporting System (AERS) was introduced to enhance postmarketing surveillance. This online database allows for the reporting of adverse effects of drugs identified by patients or prescribers.^15^

In 2000, the ClinicalTrials.gov website was made available to the public. This was mandated by the 1997 FDA Modernization Act, which required the National Institutes of Health (NIH) to improve public access to information about ongoing clinical trials. This database registers all efficacy trials conducted under an investigational new drug (IND) designation.^16^

The FDA Amendments Act of 2007 broadened and updated several previously enacted laws. This included expanding postmarketing safety activities by upgrading the AERS database and analytical tools and creating the Risk Evaluation and Mitigation Strategies (REMS) program.^17^ This program is intended to mitigate risk by allowing the FDA to require drug manufacturers to introduce additional safety provisions when the FDA has concerns about a particularly serious adverse event for a specific drug.^18^ Examples of REMS stipulations include the completion of a mandatory preadministration laboratory test if it is expected to predict the risk of an adverse event or the mandatory immediate availability of a certain treatment at the facility where a drug is being administered.

In May 2018, the Right to Try Act was signed into federal law. This allows physicians to apply for expanded access to drugs that have completed a phase I trial but which have not yet been approved by the FDA for market entrance. This law allows patients to bypass FDA approval in the pursuit of expanded access.^19^

## Development and approval process

The first step in bringing a drug to the market is the process of discovery, where many laboratory tests are carried out in order to identify a chemical compound with therapeutic potential that warrants further examination. This chemical entity is then put through further bench research and in vitro studies to evaluate its pharmacokinetics and potential for therapeutic physiologic effects. A drug showing promise will then enter the preclinical phase and undergo in vivo testing in animals primarily to assess the drug’s safety profile and determine a safe starting dose for human testing. Data are also collected at this point on the drug’s physiologic effects, pharmacodynamics, and pharmacokinetics.^20^

**Figure 3:**
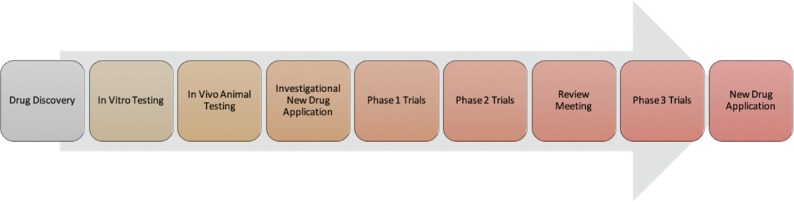
Key steps in the FDA drug approval process.

The first involvement of the FDA in the development process comes when the drug sponsor submits an IND application **(Figure 3)**. Approval must be awarded by both the FDA and the local institutional review board in order for testing in humans to commence. The primary purpose of the IND is to ensure the safety of study participants.^21^ The IND application presents information collected during preclinical testing in three broad categories: first, it offers data on the toxicity and pharmacology of the drug when administered to animals in order to confirm that, within reason, the drug can be safely administered to humans; second, it discusses the manufacturing process of the drug to allow the FDA to assess whether the manufacturer can safely produce batches of the drug with a consistent composition; and, third, it introduces the proposed clinical study protocols and the qualifications of the investigators overseeing the trials as well as presents the informed consent documents that will be provided to study participants.^22^ If the investigators are not informed that the application has been denied or that a clinical hold has been established within 30 days of submission, then phase I trials may begin. An official approval notification is not typically issued; thus, an approval can be assumed in the absence of a notification otherwise within 30 days.

Phase I clinical trials are often single-blinded controlled studies involving roughly 20 to 80 healthy volunteers. Their goal is to determine the safe dosage for human subjects and to collect data on side effects and pharmacokinetics of the drug under investigation.^23^ This information is used to help design phase II trials as well as minimize the risk to participants in those trials.^24^ Like phase I studies, phase II trials are also generally single-blinded controlled trials but instead now examine subjects with the disease for which the drug has been developed. Here, the number of included participants is typically in the hundreds, and data are collected on drug efficacy. Supplementary information is additionally compiled on drug side effects and attempts are made to determine a therapeutic dosage window wherein the efficacy and side effects are optimally balanced.^23^

The largest sample size and most informative data are collected in phase III trials. These are often double-blinded randomized controlled trials with patient numbers in the thousands. Adverse events that were not common enough to be observed in phase II trials are likely to be identified in these trials and these investigations will also be powered to allow for a statistically significant treatment benefit to be detected if the drug is sufficiently efficacious. These trials will also seek to provide information on efficacy in different patient populations as well as interactions with other drugs.^25^

Communication between the drug sponsor and the FDA is usually ongoing from the time at which the IND application is submitted; however, there are also formal meetings that generally occur before and after the phase III studies. A review meeting is conducted following the completion of phase II trials in order to discuss the data acquired thus far and to agree on design protocols for phase III trials. Following phase III trials, a pre–new drug application (NDA) meeting to discuss the imminent NDA submission is held. The NDA is the final formal request made to the FDA for drug approval and, if accepted, the drug can then be marketed in the US. Within the NDA, the drug sponsor includes all animal and human trial data with the appropriate analysis results. The FDA then must decide, on the basis of these data, whether the drug is able to provide benefits that outweigh its known or potential risks to its intended population. The FDA has 60 days to assess the completed application and formally file it for review.^23,26^ Once filed, the FDA aims to act on the NDA within 10 months. As part of this review, the FDA is also required to examine the professional labeling of the drug and inspect the facility where the drug is manufactured. An FDA approval at this stage means that the drug has met the necessary standards for safety and efficacy data, labeling, and manufacturing.

### Marketing approval

In the US, once the FDA has performed its scientific analyses and deemed safety and efficacy standards have been acceptably met, it is able to authorize market approval nationally. After this step, drug companies need only to negotiate pricing with private insurance companies or Medicaid/Medicare to generate revenue. In Europe, the scientific analysis is performed by the European Medicines Agency (EMA), an organization that is currently based in London but which is expected to relocate to Amsterdam following the United Kingdom’s withdrawal from the European Union. Following the scientific evaluations, the EMA reports their opinion to the European Commission, which can then authorize approval for a drug to be sold within the European market. In contrast with the US, following European market approval, drug companies often still must wait for further analyses to be completed prior to sale. For example, in the United Kingdom, due to a centralized health system called the National Health Service (NHS), after European market approval, drugs are then analyzed for cost-effectiveness by an organization called the National Institute for Health and Care Excellence (NICE) before the NHS will decide whether to purchase the drug and make it available to the public. This analysis requires a predetermined value to be assigned to the quality-adjusted life-years (QALY) gained through taking a certain drug. This value-based cost–benefit analysis will be used by the NHS to determine whether or not to purchase from the drug company and also as a basis for further discussion. Drug companies often face tougher negotiations on pricing when working with a large centralized buyer in comparison with smaller private payers like in the United States.^27,28^

## Approaches to approval expedition

### Orphan Drug Act

A drug is given an orphan status if it targets a disease that affects fewer than 200,000 Americans^29^ or when it is not likely to be profitable for up to seven years after approval.^30^ Anticipated sales to this limited population mean that drug companies often do not believe investment into the development of these drugs to be financially viable. After the Kefauver–Harris Amendment of 1962, the increased burden of evidence—and, therefore, the cost—required to prove drug safety and efficacy served to strengthen the economic imperative for manufacturers to pursue the development of drugs aimed at treating more common diseases so that sales would be more likely to recover the costs of development. In 1983, the Orphan Drug Act was passed^12^ in order to incentivize investment into orphan drugs. Government strategies to achieve this include the provision of tax credits, marketing rights, research grants, and development assistance. Although according to legislation, orphan drugs must achieve the same standards as all other drugs to attain FDA approval, the FDA has been shown to demonstrate more flexibility in its review of these drugs given the inherent difficulties of carrying out large clinical trials for rare diseases.^31^

### Fast-track, accelerated approval, and priority review

It was recognized that the review process for the approval of drugs treating life-threatening conditions could be reasonably abbreviated, as a greater risk is accepted when balanced against the consequences of the presence of a serious disease without therapy. In 1988, the first form of the “fast-track” process was introduced, designed to expedite the development of drugs that treat serious or life-threatening medical conditions and meet an unmet medical need (eg, no current therapy is available or the currently available therapy is inferior to the drug under development).^32^ Fast-track designation facilitates a more streamlined review process, incorporating more frequent meetings and written communications with the FDA to optimize trial design and data collection and pushing through “rolling review,” which allows each section of the NDA to be submitted for review separately rather than all sections needing to be held until the submission of the full application.^33,34^

In 1992, two more pathways for expedited approval were introduced: “accelerated approval” and “priority review.” Similar to the fast-track designation, drugs would only be eligible for these pathways if they targeted serious or life-threatening conditions and offered benefits over existing therapies. Accelerated approval allowed investigators to use surrogate endpoints rather than clinical endpoints to assess for clinical efficacy.^33^ Surrogate endpoints should be reasonably predictive of clinical benefit; commonly used surrogates in oncologic drug trials are “progression-free survival” and “time to progression.” Approving the use of surrogates can substantially shorten clinical trial duration and, therefore, the length of the development process. As the use of surrogates introduces uncertainty into the assessment of drug efficacy, in cases where accelerated approval is carried out, the FDA should require mandatory postmarket phase V trials to confirm that the surrogate markers that were the basis for drug approval do indeed predict clinical benefits such as improved mortality.^35^ Priority review was introduced in 1992 as part of the Prescription Drug User Fee Act. With this designation, the FDA aims to complete the NDA review within six months rather than within 10 months.^34^

### Breakthrough therapy

The most recent designation created to expedite drug development and approval is called “breakthrough therapy” and was initiated as part of the FDA Safety and Innovation Act of 2012.^36^ This designation has been shown to facilitate the shortest median development time of all of the FDA’s expedited programs, yet a drug must meet a higher bar in order to qualify^37^—that is, to be eligible for this program, a drug must treat a serious medical condition and the manufacturer must present preliminary clinical data suggesting that the drug offers substantial improvement in comparison with current therapies. The improvement should be based on a clinically significant endpoint, established surrogate endpoint, or safety profile. If this designation is granted, the FDA will offer all fast-track designation benefits as well as guidance on how to execute an efficient drug development program and will involve senior FDA commissioners in the organizational process.^38^ A systematic review^39^ of all approvals of breakthrough therapy drugs from 2012 to 2017 suggested that rapid approval under the breakthrough therapy pathway can sometimes occur at the expense of the strength of the overall evidence supporting the approval when compared with drugs without a breakthrough designation. From the perspective of the FDA,^40^ it is not the breakthrough designation that accounts for differing levels of evidence but rather the pragmatism that the FDA must exercise to allow testing to be “as efficient and flexible as practicable, when scientifically appropriate,”^8^ such as in the approval of drugs targeting rare diseases. As with the prior expedited programs, the postmarketing analyses in this case as well are important in confirming the benefit claims made prior to approval.

### Expanded access

Balancing early access for patients with serious illnesses that may be clinically deteriorating with ensuring that (1) therapy has been adequately tested and (2) that vulnerable patients will not be exposed to the physical and financial costs of ineffective or harmful drugs is an ongoing challenge for the FDA and the purpose of its inception.

In 1987, the FDA developed an expanded-access program in order to provide patients with an immediately life-threatening or serious condition the opportunity to apply for access to experimental therapy prior to FDA approval.^41^ For these patients to be eligible, there must be no approved therapeutic alternative and the expanded access must not compromise ongoing clinical trials.^19^ Under the FDA expanded-access program, in order for a patient to receive a drug, there would need to be a qualified prescriber, patient consent, and manufacturer agreement to supply the drug. The FDA would review and decide whether to approve based on an assessment of the risk–benefit profile from available data and as long as ongoing trials and eventual anticipated FDA approval for use in the general population would not be jeopardized.^42^

On May 30, 2018, the Right to Try bill was written into law,^43^ which allows patients to access unapproved experimental drugs without any involvement or oversight from the FDA. Patients can access an unapproved drug that has completed phase I trials provided the patient has a serious or life-threatening condition, no opportunity to participate in a clinical trial, an agreeable prescriber, and provided informed consent and there is no existing approved alternative and the manufacturer has agreed to supply the drug in question.^44^ In addition, the law does not allow the FDA to use data generated from drug use in the expanded-access population “to delay or adversely affect the review or approval of [a] drug” unless such data have critical implications.^43^

Controversy has arisen in relation to this law for the following reasons. Within the FDA expanded-access program, the prohibitive step in the process of patients gaining access to unapproved drugs has almost never been difficulty with gaining FDA approval, nor has FDA approval caused undue delays; in fact, from 2005 to 2015, the FDA approved 99.7% of requests with response times falling within an average of four days.^45^ A challenging step in successfully providing patients with access to unapproved drugs is instead persuading manufacturers to agree to provide an expanded-access supply of their drug, as many disincentives exist such as limited supplies of a drug in its developmental infancy, the administrative burden of assessing applications and tracking outcomes, a potential for promoting serious adverse clinical outcomes when administered to a sick population, and potential unfavorable influences on the timeline for full approval due to a dilution of resources.^19^ The Right to Try law does not compel manufacturers to supply their drug and, apart from preventing the FDA from integrating adverse outcomes data from the expanded-access population into the approval process, it does not address the issues that commonly dissuade drug companies from approving requests and so is therefore unlikely to significantly impact preapproval access to experimental drugs.

Despite data indicating that FDA approval has not been an obstruction to expanded access, the bill has precluded FDA involvement in the process, a step that, through a detailed risk–benefit assessment, often yields critical input that helps to protect patients such as by promoting enhanced safety monitoring or drug dosage modifications.^45^

We believe that the accelerated pathway to FDA approval is problematic. A clear distinction should be made between the mechanisms utilized to accelerate approval. In cases where the mechanism entails a concentration of knowledge and resources to improve the strategy and efficiency of the approval process to ultimately decrease approval time, such should not detract from the integrity of the process. However, if the strength of evidence for approval is compromised in the pursuit of expedited approval, this could potentially facilitate approval of a drug that could expose the public to harm. Whether or not the strength of evidence is being compromised and to what degree if so are difficult to determine, and reviews on this subject have suggested conflicting conclusions.^39,40^ Still, however, the pressure to hasten approval can conceivably lead to the acceptance of weaker evidence, and this must be guarded against at all costs.

Further, although accelerated pathways must tread a fine line between achieving maximum efficiency and ensuring adequate evidence exists to approve a therapy, we believe that the Right to Try expanded-access program represents the end of the spectrum and undermines the role of regulatory bodies. Although the argument to allow patients, especially those with life-threatening illnesses, expedited access to therapy is relevant, this law threatens to return us to an era before the FDA, when companies were able to target and exploit desperate and vulnerable sections of the public—including, for example, those with terminal illnesses—pushing products with unproven therapeutic and safety profiles. This milieu and the resultant public harm in the early 20^th^ century is what led to the establishment of the FDA in the first place.

## Cardiac safety

Although cardiac drug safety encompasses several areas including proarrhythmia risk, cardiotoxicity from oncologic drugs, noncardiac drug effects on cardiovascular outcomes, and the blood pressure effects of nonhypertensive drugs, for the scope of this review, we will focus only on the first item.

Due to observations of that rising regulatory standards and therefore increasing drug development costs and timelines were leading to a fall in the rate of new drug development, the FDA introduced a Critical Path Initiative (CPI) in 2004.^46^ The function of the CPI is to create public–private partnership consortia with the purpose of researching ways to use technology to modernize the drug development process in order to improve efficiency and productivity. One such consortium is the Cardiac Safety Research Consortium (CSRC) which, in partnership with Duke University, is a collaboration among academia, industry, and government, with the mission of supporting research specifically into the cardiac safety of medical drugs and devices. The primary goals of the CSRC are to improve the cardiovascular safety profile and enhance the developmental efficiency of medical products.^47,48^

One of the biggest focuses in the area of cardiac safety in pharmaceutical research is the proarrhythmic potential. The current bedrock for the evaluation of a drug’s proarrhythmic potential is the use of a thorough QT/corrected QT (QTc) (TQT) study, for which guidance is provided by the International Council for Harmonisation of Technical Requirements for Pharmaceuticals for Human Use (ICH), which advocates that this investigation should be part of the development process for every drug.^49^ The QT/QTc interval on a surface electrocardiogram (ECG) represents the period from ventricular depolarization to the completion of repolarization, and the prolongation of this interval has a known association with dangerous ventricular arrhythmias including, most notably, torsade de pointes (torsades). The ICH guidelines recommend that surface ECGs be performed on healthy volunteers in the early stages of clinical drug development in a controlled and randomized fashion to detect whether the drug has a QT/QTc effect of regulatory concern (ie, prolongation of approximately 5 ms). The results of the TQT study will inform the intensity of QT/QTc monitoring during the remainder of the developmental process. The overall goal of the TQT study is to detect the degree of QT/QTc prolongation that occurs with the highest therapeutic serum concentrations of the drug, and this is used as a surrogate to predict the potential of the drug to cause torsades. This technique has proven sensitive in its ability to identify proarrhythmic potential.^50^

**Figure 4:**
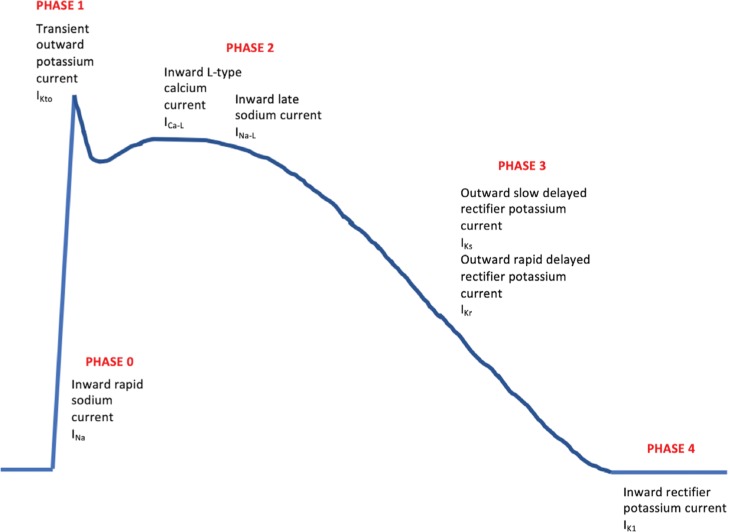
Simplified representation of a cardiac myocyte action potential.^51^

Preclinical testing for proarrhythmic risk can be used to direct modifications of molecules while in early development and as a signal for whether more comprehensive clinical testing will be required. Prolongation of the QT/QTc interval can occur through effects on any stage of the cardiac myocyte action potential **(Figure 4)**^51^; however, the outward rapid and slow delayed rectifier potassium currents (*I*_Kr_ and *I*_Ks_) appear to have the largest effect on repolarization interval. The most common way in which pharmaceutical agents delay repolarization is through the blockage of the delayed rectifier potassium channel facilitating *I*_Kr_, which is encoded by the human ether-a-go-go–related gene (*hERG*) and often referred to as the hERG current.^52^ Preclinical testing for proarrhythmic risk predominantly consists of in vitro electrophysiology studies designed to assess for the ability of a substance to inhibit the hERG current.

Criticisms of both of these methods point out their labor-intensive nature as well as lack of specificity, as both test for the QT-/QTc-prolongation potential, which is an imperfect surrogate for proarrhythmic potential. Some drugs like amiodarone and ranolazine, while capable of causing QT/QTc prolongation, have been shown to have a minimal level of proarrhythmic risk.

**Figure 5:**
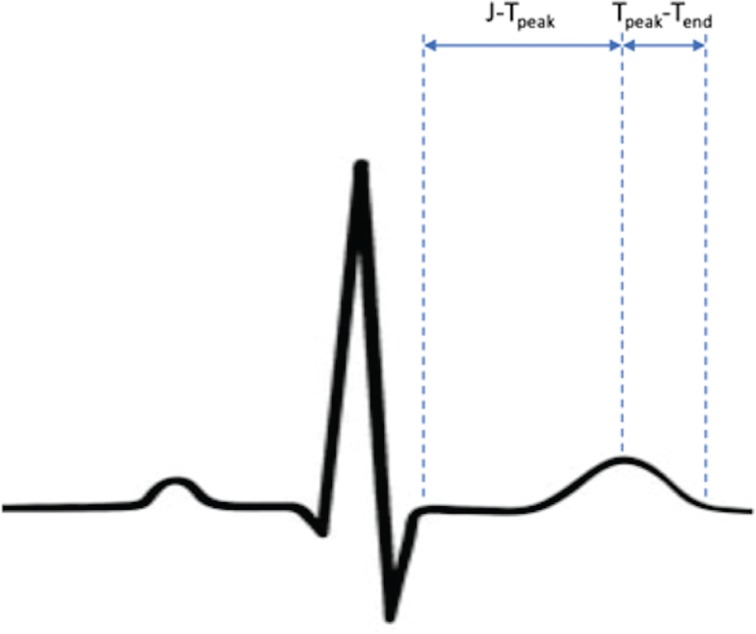
Components of the QT interval as measured during proarrhythmia risk assessment.

Although hERG current and TQT testing is sensitive and has achieved success in preventing the approval of drugs with proarrhythmic potential, the lack of specificity, which potentially has halted the development of some drugs that prolong QT/QTc but have a low torsades risk, led to efforts to devise a more precise technique to assess new drugs for their torsadogenic risk. The Comprehensive In Vitro Proarrhythmia Assay (CiPA) is a new initiative that may change the way proarrhythmia testing is carried out in the future. CiPA aims to more directly test mechanisms responsible for torsades rather than to test surrogates of torsades risk. Similarly to with the hERG current, the inward late-sodium and L-type calcium currents also contribute significantly to torsades risk. A balanced inhibition of all three currents reduces the potential for torsades provocation when compared with blockade of the hERG current alone. CiPA testing involves four components. First, ion-channel testing is carried out on human-derived models including testing of the hERG, late-sodium, and L-type calcium currents. Then, computer software is used to integrate the ion channel data into a model of human myocytes in silico and thus predict the clinical risk for torsades. Third, in vitro testing of drug effects on stem cell–derived human ventricular myocytes is performed, followed by the completion of surface ECG testing during phase I human studies.^53^ It should be noted that surface ECG testing is performed with a slightly different focus as compared with TQT testing. The ECG is assessed to specifically delineate both whether the QT/QTc interval is prolonged and also elucidate which component of it is prolonged. If both the J–T_peak_ and the T_peak_–T_end_ components **(Figure 5)** are prolonged, then this is suggestive of predominant hERG current blockade and therefore high torsades risk, whereas, when T_peak_–T_end_ is prolonged without prolongation of J–T_peak_, such is suggestive of a more balanced blockade of hERG, sodium, and calcium channels and therefore a lower torsades risk.^54^ Through CiPA assessments, a more precise evaluation of proarrhythmia is achievable, where both QT/QTc effects and clinical torsades risk can be independently assessed and reported. Collaboration among the FDA, CSRC, and other international institutions continues to advance the CiPA initiative, and a paradigm shift with regard to proarrhythmia testing is likely not far away.

## Conclusion

The job of the FDA is not only to ensure that high standards for drug efficacy and safety are applied to products made available to American consumers and patients but also to balance the lengthy, costly process of maintaining these standards against the pressure to provide access to effective treatments earlier and without surplus expenditures. Through partnerships with industry and academic institutions, research is being conducted into how information technology can be integrated into the processes of drug development to improve cost-effectiveness.
